# The Association of Insomnia, Perceived Immune Functioning, and Irritable Bowel Syndrome Complaints

**DOI:** 10.3390/jcm7090238

**Published:** 2018-08-24

**Authors:** Stephanie Balikji, Marlou Mackus, Karel A. Brookhuis, Johan Garssen, Aletta D. Kraneveld, Thomas Roth, Joris C. Verster

**Affiliations:** 1 Division of Pharmacology, Utrecht University, 3584CG Utrecht, The Netherlands; s.balikji@students.uu.nl (S.B.); M.mackus@uu.nl (M.M.); j.garssen@uu.nl (J.G.); a.d.kraneveld@uu.nl (A.D.K.); 2 Faculty of Behavioural and Social Sciences, Groningen University, 9712TS Groningen, The Netherlands; k.a.brookhuis@rug.nl; 3 Nutricia Research, 3584CT Utrecht, The Netherlands; 4 Institute for Risk Assessment Sciences (IRAS), Utrecht University, 3584CM Utrecht, The Netherlands; 5 Sleep Disorders & Research Center, Henry Ford Hospital, Detroit, MI 48202, USA; troth1@hfhs.org; 6 Centre for Human Psychopharmacology, Swinburne University, Melbourne, VIC 3122, Australia

**Keywords:** irritable bowel syndrome, sleep, insomnia, perceived immune functioning, quality of life

## Abstract

Background: Irritable bowel syndrome (IBS) can have a significant negative impact on quality of life, mood and wellbeing. The aim of this study was to investigate the association between experiencing IBS symptoms and insomnia, and perceived health status. Method: An online survey was conducted among *n* = 1950 Dutch university students (83.6% women). IBS was assessed with the Birmingham IBS Symptom Questionnaire, quality of life with the WHO-5 wellbeing index, and sleep outcomes with the SLEEP-50 questionnaire. Perceived immune functioning and general health were assessed using 1-item scales. Results: IBS symptom severity was significantly associated with insomnia complaints (r = 0.32, *p* = 0.0001), sleep quality (r = −0.21, *p* = 0.0001), sleep onset latency (r = 0.11, *p* = 0.0001) and the number of nightly awakenings (r = 0.24, *p* = 0.0001). Total sleep time was not significantly associated with IBS symptom severity. Significant correlations were also found between IBS symptom severity and perceived general health (r = −0.30, *p* = 0.0001), perceived immune functioning (r= −0.25, *p* = 0.0001), and quality of life (r = −0.24, *p* = 0.0001). Conclusions: Experiencing IBS complaints is associated with reduced perceived immune functioning, a poorer perception of general health, and sleep disturbances. These effects are reflected in a significantly lower reported quality of life in subjects with more IBS and/or sleep complaints.

## 1. Introduction

The ROME IV criteria define Irritable bowel syndrome (IBS) as a functional bowel disorder, which is characterized by recurrent abdominal pain associated with defecation or a change in bowel habits (e.g., the frequency of stool, and its appearance) [1]. The abdominal pain is accompanied by diarrhea or constipation, or both, and patients are usually categorized accordingly into IBS-C (predominant constipation), IBS-D (predominant diarrhea), and IBS-M (IBS with mixed bowel habits). If the symptoms do not clearly fit within these three categories, the diagnosis is labeled IBS unclassified. IBS the symptoms should be present for at least one day per week during the past three months, with a symptom onset of at least six months before the diagnosis is made [1]. Although the pathophysiology of IBS is unclear, it is believed that IBS complaints are related to changes in mucosal barrier integrity and secretion resulting in an increased sensitivity of the bowel, changed bowel motility, changed in cerebral-visceral perception, and the presence of psychological and physiological stressors, and food intake [2,3,4]. The worldwide prevalence of IBS is 11.2% and IBS is most prevalent among women [5]. Similar prevalence rates are reported for The Netherlands, USA and Australia [6,7,8].

Recent research has shown that IBS can have a significant negative impact on quality of life and wellbeing [9,10,11]. Although IBS has a high prevalence and a wide range of problems, it has an underestimated economic impact in terms of reduced productivity, and reduced daytime alertness on the performance of daily activities [9,11], such as driving a car and job performance. Activities affecting quality of life that are most affected in patients with IBS comprise exercise, socializing, and walking [12]. Given this, more research into factors that contribute to the presence and severity of IBS is warranted. Despite its prevalence, there is currently no effective cure, and treatments vary from medicinal symptom management to dietary interventions, and psychotherapeutic approaches [1,6].

Diagnosis of IBS requires a clinical evaluation of the history and current presence of IBS symptoms and a physical examination to exclude organic etiology. If warranted, blood tests may be conducted to exclude other diseases [1]. Alternatively, there are several instruments developed to screen for the possible presence of having IBS, or to assess the severity of IBS-related complaints.

The use of these patient-reported outcome measures (PROMs) is not solely effective for research purposes to identify disease correlates. The US Food and Drug Administration (FDA) supports the use of PROMs to determine a patient’s health status as they may support labelling claims in medicinal drug development [13]. In addition, in a clinical setting PROMs may help identifying the patient’s primary disease-related concerns. Furthermore, aligning the clinician’s focus with the patient perspective may significantly improve both treatment efficacy and patient satisfaction [14]. The ROME I, II, and III trial committees have consistently stated that the primary outcome in IBS trials should be a patient-reported outcome measures. As such, the committees strongly underlined the need for developing sensitive assessment tools.

An example of such instrument is the Birmingham IBS Symptom Questionnaire [15]. The aim was to develop a symptom score suitable for use in therapeutic trials in IBS, i.e., a scale that can reliably assess (changes) in IBS symptom severity. The Birmingham IBS Symptom Questionnaire has been applied in several scientific studies, ranging from studies demonstrating the association between quality of life and IBS symptom severity [12], dietary intervention studies demonstrating an association between dietary intake of fructose and lactose and IBS pain symptoms [16], to clinical trials examining the efficacy of treatments in reducing IBS symptoms [17,18].

A commonly reported association with having gastrointestinal functional disorders is experiencing co-occurring sleep problems [9,10,11,19,20,21,22]. An explanation for the association between IBS and sleep may be found in a mediating role of low grade inflammation. Immunological alternations have also been demonstrated in several gastrointestinal functional disorders such as IBS [11].

Pro-inflammatory cytokines such as tumor necrosis factor α (TNFα), interleukin (IL)-6, IL-8 and IL-17 have been shown to be elevated in IBS patients, as compared to healthy controls. Furthermore, anti-inflammatory cytokines such as IL-10 have been shown to be decreased in IBS patients. This suggests an important role of serum level cytokines as immune mediators in the development of IBS [23,24]. However, much remains unclear about the relationship between sleep, immune functioning and IBS. Although objective assessments of sleep and immune status are important, they do not necessarily correlate with perceived immune and health status, or sleep quality [25]. People only seek medical care, or consider health and lifestyle changes, when they subjectively experience negative health effects, such as sleep disturbances or reduced immune functioning [26]. Therefore, it is essential to also acquire PROMs relating their perceived immune functioning and perceived sleep quality.

The present study aimed to evaluate to what extent IBS symptoms are associated with sleep complaints, perceived immune functioning and general health perception. It was hypothesized that people with IBS complaints experience more sleep complaints, and report significantly more often having reduced immune functioning, and have a poorer perception of their general health. This would then be reflected in lower quality of life ratings.

## 2. Materials and Methods

Dutch university students, aged 18 to 30 years old, were recruited to complete an online survey on food and health. The survey was designed by using www.surveymonkey.com. The survey was advertised via the online social platform www.facebook.com during autumn 2016. The University of Groningen Psychology Ethics Committee approved the study. Online informed consent was obtained from all participants before starting the survey.

### 2.1. IBS Complaints

The presence and severity of IBS symptoms were determined with a modified version of the Birmingham IBS Symptom Questionnaire [15]. For the purpose of this study, the English language version of the Birmingham IBS Symptom Questionnaire was translated into a Dutch language version, and modified to improve readability. The questionnaire consists of 11 items, with 6-answer possibilities, ranging from 0 (‘none of the time) to 5 (‘all of the time’). The sum-score of the 11 items, ranging from 0 to 55 points, represents overall IBS symptom severity. Higher overall IBS scores indicate a greater likelihood of the diagnosis IBS. In addition, scores on three symptom specific scales representing the factors ‘diarrhea’ (5 items), ‘constipation’ (3 items), and ‘pain’ (3 items) were calculated. Previous research showed that the subscales of the English language Birmingham IBS Symptom Questionnaire has an acceptable reliability (Cronbach’s alpha 0.74–0.90), had good test-retest reliability, and scores correlated significantly with IBS-related quality of life assessments [16].

### 2.2. Sleep Disturbance and Sleep

The presence and severity of past month sleep disturbances were examined by the insomnia subscale SLEEP-50 questionnaire [27]. The insomnia subscale consists of nine items, which can be scored on a 4-point scale: 1 (not at all), 2 (somewhat), 3 (rather much) and 4 (very much). Cut-off value for a positive screen for insomnia is ≥19 (sensitivity = 71%, specificity = 75%). In addition to the insomnia subscale, participants had to record their past month average time to bed, time of start sleeping, and wake up time. This data were collected to compute total sleep time (TST), and sleep onset latency (SOL). The number of nightly awakenings was also recorded. Finally, participants rated their overall sleep quality on a scale ranging from 0 (very bad) to 10 (very good). In recent research, the insomnia subscale score successfully differentiated healthy control subjects from those with reduced perceived immune functioning [26], and those who screened positive for eating disorders from subjects with healthy eating habits [28].

### 2.3. Perceived Immune Functioning and Perceived Health Status

Perceived immune functioning and perceived health status were assessed using single item questions [19]. The questions were scored from 0 (very poor) to 10 (excellent). In addition, participants were asked whether they experienced reduced immune functioning at the moment of completing the survey (a yes-no question). Previous research showed that 1-item perceived immune functioning and general health scores correlated significantly with mental resilience [29], autism traits [30], and the Immune Function Questionnaire [29].

### 2.4. Quality of Life

Quality of life was measured with the 5-item World Health Organization (WHO-5) Well-Being Index [31,32]. Items are scored on a 5-point Likert scale ranging from 0 (“At no time”) to 5 (“All of the time”). The raw score, ranging from 0 to 25, is multiplied by 4 to achieve a final score. Higher scores represent better wellbeing/quality of life over the past two weeks. Cronbach’s alpha of the WHO-5 is 0.82 [31]. Previous research showed that scores on the WHO-5 significantly correlated with mental health (e.g., depression scores) and psychological constructs such as mental resilience and self-esteem [29,31].

### 2.5. Statistical Analyses

Statistical analyses were conducted by using IBM SPSS Statistics for Windows, Version 24.0 (IBM Corp., Armonk, NY, USA). Nonparametric Spearman’s rho correlations were used to examine associations between the assessed variables. Correlations were considered significant if *p* < 0.05.

## 3. Results

*n* = 1950 subjects (83.6% women) completed the survey and were included in the analyses. Their mean (SD) age was 21.3 (2.1) years old. Characteristics of the study population are summarized in Table 1. It is evident from Table 1 that men and women differed significantly on most variables. Hence, the analyses presented in this paper will address first the effects observed in the population as a whole, followed by the same analyses conducted for men and women separately.

### 3.1. Positive Vs. Negative Screen for Insomnia

The mean (SD) score on the SLEEP-50 insomnia subscale was 16.4 (5.3). Twenty-nine percent of the participants (*n* = 572) screened positive for insomnia (mean insomnia score 23.3 vs. 13.5 for those who screened negative for insomnia). As expected, when compared to those who screened negative for insomnia, they reported significantly (*p* < 0.0001) lower sleep quality (7.4 vs. 5.5), significantly longer SOL (16.8 vs. 33.6 min), and significantly more nightly awakenings (0.6 vs. 1.6). No significant difference was observed in TST. When compared to those who screened negative for insomnia, those who screened positive for insomnia reported significantly (*p* < 0.0001) more overall IBS complaints (9.7 vs. 13.6), and had significantly higher scores on the subscales constipation (3.0 vs. 4.2), diarrhea (3.7 vs. 4.9), and pain (3.0 vs. 4.5).

### 3.2. Perceived Reduced Resistance Vs. Normal Immune Status

About one third of the participants (33.5%, *n* = 654) reported reduced perceived immune resistance. When compared to those who reported a normal immune status, they reported significantly (*p* < 0.0001) lower sleep quality (7.0 vs. 6.5), significantly longer SOL (20.8 vs. 23.6 min), and significantly more nightly awakenings (0.9 vs. 1.1). No significant difference was observed in TST. When compared to those who reported a normal immune status, those who reported reduced immune resistance reported significantly (*p* < 0.0001) more overall IBS complaints (9.9 vs. 12.7), and had significantly higher scores on the subscales constipation (3.1 vs. 3.9), diarrhea (3.7 vs. 4.8), and pain (3.1 vs. 4.1).

### 3.3. Associations

The observed Chronbach’s alpha for the Dutch IBS scale was 0.83, and Chronbach’s alpha for the subscales on ‘diarrhea’, ‘constipation’, and ‘pain’ were 0.66, 0.83, and 0.77, respectively. Statistically significant correlations were found between IBS scores and insomnia (r = 0.32, *p* = 0.0001), sleep quality (r = −0.21, *p* = 0.0001), SOL (r = 0.11, *p* = 0.0001), and number of nightly awakenings (r = 0.24, *p* = 0.0001). No significant association was found between IBS scores and TST (r = 0.01, *p* = 0.666). Significant correlations were also found between IBS scores and perceived general health (r = −0.30, *p* = 0.0001), the 1-item perceived immune functioning (r = −0.25, *p* = 0.0001), and quality of life (r = −0.24, *p* = 0.0001). Similar significant correlations were observed for the three IBS subscales, of which those with the IBS—pain subscale was most strong (data not shown). Table 2 gives an overview of all correlations.

Correlations of sleep variables with IBS in men were comparable to those seen in women. An exception was the number of nightly awakenings, which correlated significantly with IBS scores in women (r = −0.23, *p* = 0.0001), but not in men (r = 0.10, *p* = 0.072). Similarly, all IBS subscales correlated significantly with the number of nightly awakenings in women, whereas in men only the IBS subscale ‘pain’ showed a significant association. Furthermore, men showed stronger correlations than women between IBS and perceived general health (r = −0.39 vs. r = −0.28, respectively) and perceived immune functioning (r = −0.25 vs. r = −0.22, respectively).

## 4. Discussion

The current study demonstrated the robust association between having irritable bowel syndrome symptoms and sleep complaints. Although no significant association was found between IBS scores and TST, subjects reported higher total IBS scores scored significantly higher on the SLEEP-50 subscale of insomnia, sleep quality, SOL and the number of nightly awakenings. Therefore, in subjects with more IBS complaints sleep was more fragmented and of poorer quality. It was further shown that perceived immune functioning is associated with IBS and sleep complaints, which are reflected in a reduced general health perception and lower quality of life.

Our findings are supported by previous studies [33,34,35,36,37,38]. For example, Rotem et al., reported that patients with IBS have impaired sleep quality, reduced slow-wave sleep activity and significant sleep fragmentation [33]. Using the Birmingham IBS Symptom Questionnaire, a recent study by Lee et al., showed significant associations between sleep disturbances and IBS related complaints in patients with endometriosis [38]. In particular, the severity of IBS pain complaints correlated significantly with trouble falling asleep, the need for sleep medication, being awakened by pain during the night, being awakened by pain in the morning, and overall sleep quality. These association were not significant for IBS related constipation or diarrhea. Ali et al., have pointed at the involvement of both sleep and the immune system in the development of several gastrointestinal disorders [11]. In this context, increases in blood concentrations of inflammatory cytokines such as TNF-α, IL-1 and IL-6 have been associated with experiencing sleep complaints [11,34,35,36,37]. And vice versa these studies showed that sleep complaints may alter the expression of these inflammatory cytokines. For example, in subjects with reduced immune functioning or chronic inflammatory diseases sleep complaints are commonly reported.

It is important to understand the relationship between IBS complaints, sleep and perceived immune functioning. Both good sleep quality and a well-balanced immune function are necessary to achieve positive health outcomes. Given this, individuals with IBS complaints could benefit from improving sleep quality and immune functioning. This could be established for example by adopting effective sleep hygiene habits, or changing dietary intake in order to promote a healthy immune status (e.g., more vitamins and fibers, and less fat). The corresponding reduction in IBS symptoms would then increase wellbeing and quality of life.

It is evident from the current study that both IBS symptoms and sleep disturbances have a negative impact on quality of life. Previous research also showed that IBS symptoms may negatively impact various factors associated with quality of life, including basic activities such as exercise and walking, but also interpersonal outcomes such as, socializing were negatively affected [12]. Given this, the possible effects on daily activities such as job performance and driving warrant further investigation.

Some limitations should be considered when interpreting the current findings. First, our study population consisted of students, aged 18 to 30 years old, which limits generalizability to the population at large. Moreover, the lifestyle of a student may play an essential role in the prevalence of IBS symptoms and sleep complaints. Students more often have irregular sleep patterns that peer who has a job and more regular lifestyle. Furthermore, factors, such as diet, transition from living in a family setting to student life, alcohol intake, and academic stressors may differ between students and individuals belonging to the same age group but having a regular nine-to-five job. Secondly, women were overrepresented in the study population of this survey, reflecting the gender distribution at the universities in The Netherlands. It should be noted that IBS has a higher prevalence in women than men in The Netherlands [1]. However, the number of participating males was sufficiently large to conduct statistical analyses for men and women separately. Thirdly, the population under investigation was young and relatively healthy. Only a minority (29.3%) of participants scored positive on the insomnia subscale, and most reported a relative healthy immune functioning. The study should therefore be replicated in patients that are formally diagnosed with insomnia to determine if comparable results will be observed. Given this, we did find significant associations between IBS complaints, sleep, and perceived immune functioning. The fact that these associations are present already in relative healthy subjects strengthens the hypothesis that in IBS patients these associations are even stronger. Fourthly, the Birmingham IBS Symptom Questionnaire was not specifically designed for diagnostic purposes. It provides severity scores on IBS related symptoms of pain, diarrhea, and constipation but the scale does not have cut-off values for screening positive or negative for having IBS. As a formal diagnosis is required to establish whether someone has IBS, it is unsure to what extent participants of the current sample should be diagnosed as such or not. Finally, all assessments in this study were self-reported. Future studies should confirm these findings using objective assessments of sleep (e.g., polysomnography) and immune functioning (e.g., determining blood cytokine concentrations). Preferably such a study should compare subjects that are formally diagnosed with IBS and/or insomnia, and compared to a healthy matched control group.

## 5. Conclusions

Taken together, the findings of the current study show clear associations between IBS complaints, sleep quality, and perceive immune functioning and wellbeing. Further evaluation of these associations and interventions to modulate them, such as sleep hygiene adaptations and dietary or lifestyle interventions to improve immune functioning, are necessary to improve the wellbeing and quality of life of patients suffering from IBS.

## Figures and Tables

**Table 1:** Characteristics of the study population.

	Overall	Men	Women
	*n* = 1950	*n* = 319	*n* = 1631
Age (years)	21.3 (2.1)	21.7 (2.1)	21.2 (2.1) *
BMI (kg/m^2^)	22.4 (3.2)	23.1 (2.9)	22.2 (3.2) *
IBS—overall	10.8 (7.1)	7.3 (5.2)	11.5 (7.2) *
IBS—constipation	3.3 (3.0)	2.2 (2.0)	3.6 (3.1) *
IBS—diarrhea	4.1 (3.1)	3.3 (2.6)	4.2 (3.2) *
IBS—pain	3.4 (2.8)	1.8 (1.9)	2.8 (0.1) *
Perceived Immune functioning	7.5 (1.4)	8.0 (1.3)	7.4 (1.4) *
Perceived health	7.4 (1.1)	7.6 (1.1)	7.4 (1.1) *
Quality of life	52.3 (15.9)	55.5 (16.5)	51.7 (15.8) *
Insomnia	16.4 (5.3)	15.4 (4.7)	16.6 (5.4) *
Total sleep time (min)	519.9 (65.4)	507.9 (72.4)	522.2 (63.8) *
Sleep onset latency (min)	21.7 (15.6)	20.6 (14.4)	22.0 (15.8)
Sleep quality	6.9 (1.5)	6.9 (1.5)	6.9 (1.5)
Number of nightly awakenings	0.9 (1.1)	0.6 (0.9)	1.0 (1.1) *

Mean (SD) values are shown for each variable. Significant differences (*p* < 0.05) between men and women, as observed applying an independent samples Mann-Whitney U Test, are indicated by *. Abbreviations: BMI = body mass index, IBS= irritable bowel syndrome.

**Table 2:** Associations between IBS, perceived health and immune status and sleep variables.

	(1)	(2)	(3)	(4)	(5)	(6)	(7)	(8)	(9)	(10)	(11)
IBS—overall (1)	-----										
IBS—constipation (2)	0.74 *	-----									
IBS—diarrhea (3)	0.80 *	0.38 *	-----								
IBS—pain (4)	0.81 *	0.44 *	0.53 *	-----							
Perceived immune functioning (5)	−0.25 *	−0.16 *	−0.20 *	−0.24 *	-----						
Perceived health (6)	−0.30 *	−0.21 *	−0.26 *	−0.26 *	0.57 *	-----					
Quality of life (7)	−0.24 *	−0.16 *	−0.17 *	−0.24 *	0.26 *	0.41 *	-----				
Insomnia (8)	0.32 *	0.23 *	0.23 *	0.30 *	−0.19 *	−0.28 *	−0.41 *	-----			
Total sleep time (9)	0.01	−0.01	0.02	−0.01	−0.06 *	−0.01	0.01	−0.02	-----		
Sleep onset latency (10)	0.11 *	0.07 *	0.06 *	0.11 *	−0.12 *	−0.16 *	−0.23 *	0.56 *	0.08 *	-----	
Sleep quality (11)	−0.21 *	−0.16 *	−0.14 *	−0.20 *	0.19 *	0.31 *	0.40 *	−0.72 *	0.07 *	−0.49 *	-----
Nightly awakenings (12)	0.24 *	0.19 *	0.15 *	0.25 *	−0.10 *	−0.17 *	−0.18 *	0.49 *	0.03	0.26 *	−0.37 *

Significant nonparametric Spearman’s r correlations (*p* < 0.05) are indicated by *. Abbreviations: IBS, irritable bowel syndrome.
